# Tinkering at the margins: evaluating the pace and direction of primary care reform in Ontario, Canada

**DOI:** 10.1186/s12875-019-1014-8

**Published:** 2019-09-11

**Authors:** Monica Aggarwal, A. Paul Williams

**Affiliations:** Institute of Health Policy, Management, and Evaluation, University of Toronto, 155 College Street, Suite 425, Toronto, ON M5T 3M6 Canada

**Keywords:** Primary care, Primary health care, Primary care reform, Primary care models, Evaluation, Core dimensions

## Abstract

**Background:**

Primary care reform has been on the political agenda in Canada and many industrialized countries for several decades; it is widely seen as the foundation for broader health system transformation. Federal investments in primary care, including major cash transfers to provinces and territories as part of a 10-year health care funding agreement in 2004, triggered waves of primary care reform across Canada. Nevertheless, Commonwealth Fund surveys show, Canada continues to lag behind other industrialized nations with respect to timely access to care, electronic medical record use and audit and feedback for quality improvement in primary care. This paper evaluates the pace and direction of primary care reform as well as the extent of resulting change in the organization and delivery of primary care in Ontario, Canada’s most populous province.

**Methods:**

Qualitative and quantitative methods were used for this study. A literature review was conducted to analyze the core dimensions of primary care reform, the history of reform in Ontario, and the extent to which different dimensions are integrated into Ontario’s models. Quantitative data on the number of family physicians/general practitioners and patients enrolled in these models was examined over a 10-year period to determine the degree of change that has taken place in the organization and delivery of primary care in Ontario.

**Results:**

There are 11 core reform dimensions that individually and collectively shift from conventional primary care toward the more expansive vision of primary health care. Assessment of Ontario’s models against these core dimensions demonstrate that there has been little substantive change in the organization and delivery of primary care over 10 years in Ontario.

**Conclusions:**

Primary care reform is a multi-dimensional construct with different reform models bundling core dimensions in different ways. This understanding is important to move beyond the rhetoric of “reform” and to critically assess the pace and direction of change in primary care in Ontario and in other jurisdictions. The conceptual framework developed in this paper can assist decision-makers, academics and health care providers in all jurisdictions in evaluating the pace of change in the primary care sector, as well as other sectors.

## Background

More than 40 years after the groundbreaking Declaration of Alma-Ata identified primary health care (PHC) as “essential health care” [1], there is substantial consensus across the industrialized world that improvements in “first contact” health care are crucial not only to enhance population health but to sustain increasingly stretched health care systems. PHC that includes, but goes beyond a “narrow offer of specialized curative care” to embrace health promotion and the determinants of health, continues to promise “better health, less disease, greater equity, and vast improvements in the performance of health systems” [2]. Even when it does not fully attain this expansive vision, high-performing primary care (PC) is widely recognized as the foundation of an effective and efficient health care system. There is ample evidence that countries with strong PC have demonstrably better health outcomes and health equity as well as lower mortality rates and overall costs [3–5].

In spite of successive waves of primary care reform (PCR) in Canada aimed at strengthening PC, and advancing toward PHC, actual change in its organization and delivery has been modest. In Ontario, Canada’s most populous province, a variety of PCR models have been implemented over decades. Nevertheless, much of PC continues to be provided to individual patients, by individual doctors, working in private practice on a fee-for-service (FFS) basis [6], often without after-hours arrangements or formal connections to other health care providers [7].

To analyze the pace and direction of change in the organization and delivery of PC in Ontario and other jurisdictions, and particularly, to understand what progress has been made as a result of policy reforms, it is necessary to understand two key points. First, PCR is not a monolithic project. Different reform models constitute multi-dimensional “bundles” of elements that move more or less some distance from conventional physician-led PC to more expansive conceptualizations of PHC. While, for instance, some reforms may aim to alter physician payment, more ambitious reforms may also look to achieve broader population health goals such as; organizing services to meet the needs and expectations of individuals and communities; and building healthier communities through sector integration [2].

Second, because PCR is about change, “transforming the way the health care system works today” [8], it will challenge, or at least push the limits of hard fought political bargains at the foundations of health care systems [9]. In Canada, such bargains, negotiated historically and often in conflict between governments and the organized medical profession, have been characterized in terms of “private practice, public payment” [10]. While under Canada’s health insurance system (Medicare) public government pays for universal access for medically-necessary hospital and doctor services, control over delivery still rests largely with private providers. Physicians retain considerable scope to determine not only what constitutes medical necessity, but how they run their practices, including location, hours, services, and who they will accept as patients. As a result, reforms which seek to change the way in which PC is organized and delivered will almost inevitably challenge such bargains to a lesser or greater degree. For example, reforms that aim to move from physician-led FFS practice to community-led salaried clinics will likely encounter more resistance than reforms aimed at ensuring that “orphan patients” gain access to family physicians/general practitioners (FPs/GPs) by enhancing professional fee schedules. When assessing the pace and direction of reform and change, it is essential both to “unpack” the different dimensions of reforms and to analyze the extent to which they challenge historical bargains underlying health systems.

A first key objective of this research is to identify the fundamental “core dimensions” of PCR aimed at strengthening PC and moving toward the more expansive vision of PHC. The second objective is to examine the characteristics and history of PCR models in Ontario and the extent to which different core dimensions are integrated into these models. The third objective is to examine data on the degree of FP/GP participation in different PCR models and the “shares” of patients served by each model in Ontario. To date, there is a paucity of literature that examines how the core dimensions of PCR can be used to evaluate the pace and direction of the PC sector.

## Methods

This study employed a mix of qualitative and quantitative methods. It was guided by a theoretical framework based on neo-institutional theory from the policy sciences that highlights how decisions made at key historical junctures shape subsequent decisions and “policy pathways” going forward; as detailed elsewhere, this theory also draws attention to the role played by powerful societal actors, such as the organized medical profession, in influencing these decisions [11].

The qualitative study involved a literature review on the core dimensions of PCR and the historical development of PCR in Ontario. Since this study is a follow-up of previous research on PCR [11], the study period was June 2008 to June 2018.

The literature review was conducted by identifying both published and grey literature in the following databases: MEDLINE, the Cumulative Index to Nursing and Allied Health Literature, EMBASE and Scopus. Grey literature search tools included: Google, Google Scholar, Scirus, and Yahoo. The search strategy that was used to retrieve papers and reports involved combining truncated keywords. The first search combined: *‘dimension’ OR ‘attribute’* AND ‘*primary care’* OR ‘*primary health care*’ OR ‘*primary care reform’* OR ‘*primary health care reform’*. The second search combined: ‘*history*’ AND “*primary care’* OR ‘*primary health care’* OR ‘*primary care reform’* OR ‘*primary health care reform’* AND ‘*Ontario*’. The titles and abstracts of all citations retrieved from the search were screened against the following study inclusion criteria: dimensions PCR, history of PCR in Ontario, papers published in English, and documents from 2008 to 2018. As part of the search strategy, additional efforts were made to obtain documents (published, grey literature and unpublished) related to PCR in Ontario, including planning documents, policy documents, position papers from various organizations and interest groups and contractual agreements in Ontario. Titles were selected according to the relevance of the abstracts to the topic. Reference sections of each publication were hand-searched to identify relevant articles. Full text was obtained for titles and abstracts that fulfilled the inclusion criteria. The screening process resulted in the identification of 95 relevant publications. Deductive and inductive coding was done of each publication to identify the core dimensions of PCR. The data was extracted, compiled and analyzed to identify the core dimensions of PCR that were most frequently identified in the literature.

Informed by our neo-institutional theoretical framework, we analyzed all documentation related to the history of PCR in Ontario over the 10-year period (2008–2018) to reconstruct the sequence of key events and situate them in their context, highlight critical decisions and the reasons for these decisions, examine the role of societal actors, and assess the extent to which different reform dimensions are integrated into Ontario’s PCR models [12].

Quantitative data on the number of FPs/GPs and patients enrolled in PCR models was obtained from Ontario’s Ministry of Health and Long Term Care.

This study will make an important contribution to the literature by developing and elaborating a conceptual framework that can be used to examine the extent to which change takes place in the organization and delivery of PCR in a single jurisdiction such as Ontario, or comparing the pace and direction of change in different jurisdictions.

## Results

This paper is presented in three parts. In the first part, we provide a brief introduction to Canada’s health care system, the political and institutional context for PCR in Ontario. In part two, we identify fundamental “core dimensions” of PCR that are most frequently identified in the PCR literature. The third part provides an overview of the history of PCR models in Ontario and the extent to which different core dimensions are integrated into these models; we then present data showing the degree of FP/GP participation in different PCR models and the “shares” of patients served by each model in Ontario.

### Part 1: Canada’s health care system (Medicare)

Canada’s health care system is not, as the name might suggest, a health care delivery system. Rather it is a publicly funded, universal health insurance plan that pays for a relatively narrowly defined “basket” of medical services. Because Canada’s 13 provincial/territorial (P/T) governments have primary jurisdiction over health care, each administers its own version of the plan, funded in part by the federal (national) government which uses its “spending power” to ensure that P/T plans meet minimal federal terms and conditions [13]. To be eligible for full federal health funding, P/T governments must provide universal access to “medically necessary” services provided in hospitals or by doctors. P/T governments can choose to provide coverage for additional services such a prescription drugs and home care, which all do, although coverage varies widely (e.g., in Ontario, public coverage for prescription drugs is mainly for seniors, persons with low incomes and children and young adults under age 25).

While approximately 70% of all health care *financing* in Canada is through public tax revenues [13], health care *delivery* remains mostly through private for-profit, private not-for-profit and public organizations. For example, most Canadian PC physicians continue to work in private practices, essentially small businesses [6]. Although they bill publicly-funded Medicare for the insured services they provide, most physicians remain private autonomous entrepreneurs with considerable control over the content and conditions of their medical work. This arrangement, based on an historical bargain between government and the medical profession at the time of Medicare’s inception, results in minimal accountability for the delivery of medical care [9, 10, 14]; the policy focus remains on the payment side. Even then governments have limited scope to act. While negotiation of global physician budgets takes place directly between the P/T governments and their respective medical associations, most fees for specific medical services are set by the associations themselves and individual physicians largely determine how they organize and deliver care incuding the range of services they provide, where and when they provide them, and who they provide them to. Even where P/T governments have established regional entities to coordinate and integrate health care at the local level, these entities have not been ceded direct authority over physician services. Nunavut and Northwest Territories, two northern territories with small populations, are the only Canadian jurisdictions where physicians are under direct contract with the government [15, 16].

In the early 2000s, converging factors pushed PCR high onto the health policy agenda across Canada. These included the findings and recommendations of numerous national and provincial reviews pointing to gaps in “first contact” care and the need for PCR; growing political and public concern about health care access and quality; mounting dissatisfaction among FPs/GPs with their working conditions and their ability to provide high-quality care to patients with increasingly complex needs; and medical school graduates’ declining interest in family medicine [17]. In his influential 2002 *Report on the Future of Health Care in Canada*, Romanow commented on the “high degree” of accord around the importance of developing a complete and effective PC system that can ensure: continuity and coordination of health services to promote individual and population health; 24/7 access to care; early detection and action; and information on needs and outcomes. Similarly, the Canadian Senate report on *The Health of Canadians,* led by Michael Kirby [18], advocated for a shift to multi-disciplinary teams; providing comprehensive services including health promotion and disease prevention on 24/7 basis; adoption of alternative methods of funding; and full integration of electronic health records.

Massive new public health care investments provided additional impetus for reform. In 2001, the First Ministers (including the Prime Minister of Canada and P/T Premiers) established an $800 million Primary Health Care Transition Fund to support PCR demonstration projects as well as research [17]. The 2003 First Ministers Health Accord provided $16 billion additional federal dollars in a Health Reform Fund targeting PHC, home care, catastrophic drug coverage and hospital wait times. In 2004, the First Ministers established a goal of 50% of Canadians having *24/7 access to multidisciplinary PHC teams* by 2011, and they agreed to “*accelerate the development and implementation of the electronic health record*.” [17].

To achieve this goal P/T governments have relied heavily on “exhortation” and “expenditure,” in effect, trying to convince PC physicians to alter the way in which they practice through persuasion or economic incentives; participation by PC physicians in reform has been solely on a voluntary basis. Moreover, when PCR funding has been committed, it has almost always been *in addition* to negotiated budgets for physician services. In Ontario, for example, reforms have relied heavily on financial incentives to persuade PC physicians to adopt reform models. This creates a new dilemma: rather than generating economic efficiencies or controlling costs, PCR initiatives almost always result in added costs at least in the short run, making successive governments hesitant to engage in large-scale reform particularly in the absence of hard evidence showing that incentives do more than increase physicians’ incomes [19].

Progress has been mixed. On the one hand, Canadian jurisdictions have implemented a constellation of PCR models, with details always painstakingly negotiated with medical associations. Yet few reform models have incorporated more than sub-sets of core reform dimensions such as alternatives to fee-for-service (FFS) payment, interdisciplinary teams, and a population health focus [11, 20], seen by the WHO and others to ensure high performing PC systems [20–24]. Perhaps not coincidentally, PC performance in Canada continues to lag behind ten other developed countries in areas including timely access to care, after-hours care, electronic medical record (EMR) use and audit and feedback for quality improvement (QI) [25].

### Part 2: PCR “Core” dimensions

In our previous work, we conducted an extensive review of the international literature to identify nine “core” dimensions of PCR [11], elements seen to be essential to strengthening PC. These included a population health approach; group practice setting; the use of inter-professional teams; alternatives to FFS; enrolled or rostered patients; patient and community engagement; 24/7 access to care; the use of information technology (IT); and system coordination and integration.

**Fig. 1:**
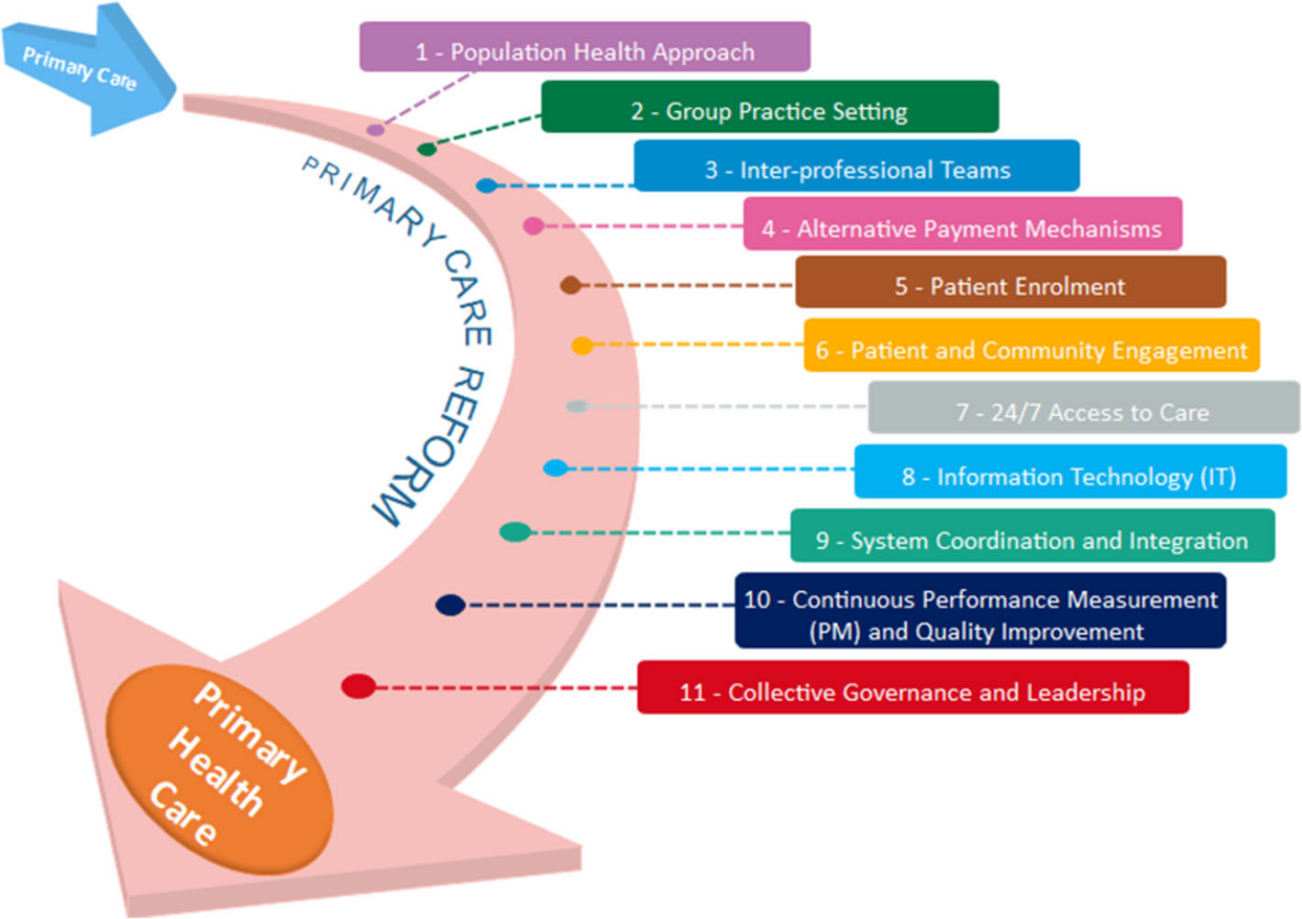
Core Dimensions of PCR

Our more recent review of the literature used the same approach from our previous work and identified two additional core dimensions: continuous performance measurement (PM) and quality improvement (QI) [17, 26, 27]; and collective governance and leadership [26, 28–31]. These, together with our original nine core dimensions, are illustrated in Fig. 1 and explained below. It is important to note that these dimensions are those that are most frequently identified in the PCR literature, which reflect movement from PC towards PHC, and do not fully encompass the expansive vision of PHC, as defined by the WHO.

In addition, a key point to keep in mind is that some or all of these dimensions may be “bundled” in different reform “models.” While, as we shall see, some Ontario models incorporate most of the 11 identified “core” dimensions, others incorporate only one or two. Although reality is often complex, a good rule of thumb is that the greater the number of core dimensions incorporated into particular reform models, the greater their potential for change in the organization and delivery of PC. Of course, because they move farther away from Medicare’s historical bargain, high change models will also be inherently harder to achieve.

#### Dimension 1: population health approach

A first core dimension of reform focuses on a shift from a relatively “narrow” interpretation of PC as expert-driven curative care, to a broader remit including health promotion and the determinants of health, the social, economic, environmental and biological factors impacting on individual and population health [32, 33]. Progress along this core dimension involves an increasing emphasis on health and wellness and includes disease prevention, health promotion, and the social determinants of health (income and social status (e.g., poverty reduction); physical environment (e.g., improving water or air quality); and education and literacy (e.g., for immigrants)) [34, 35]. A recent review indicates the core elements of a population health approach include*:* population orientation, understanding needs and solutions through community outreach, addressing health disparities/health in vulnerable groups, and inter-sectoral action and partnerships [36]. In practice, PC providers would identify the health needs of the patient population based on geographical location, and determine how best to meet those needs [37, 38]. This can be through inclusion of services within a single organization or by coordinating across multiple health and social care organizations.

#### Dimension 2: group practice setting

A second dimension looks to the extent to which physicians collaborate around patient care. While maximizing physician autonomy and control over clinical and financial aspects of practice, solo PC practice also constrains scope of services and hours, and the ability to seek advice from peers. By contrast, PHC favors collaborative group settings [17, 39] supporting peer support and care continuity. Change along this core dimension involves movement from solo to forms of group practice which may include geographic co-location or virtual collaboration through information technology. Here, it is important to distinguish between groups that provide shared clinical care and those that are essentially business partnerships with no shared care.

#### Dimension 3: inter-professional teams

The third dimension entails movement even beyond groups of physicians to inter-professional teams [40, 41]. By combining the knowledge and expertise of different professions (such as nurses, nurse practitioners, therapists and technologists), inter-professional teams can facilitate the provision of more comprehensive, continuous and person-centred care, more effectively mobilize health care resources and assist with patient navigation of the health care system. Teams can be involved in care management activities such as patient education, medical management and adherence support, risk stratification, population management, coordination of care transitions and care planning [37]. Inter-professional teams can also achieve efficiencies by allowing team members to function at the top of their skill set (or “scope of practice”) and ensuring that there is a clear understanding of roles and responsibilities [20].

#### Dimension 4: alternative payment mechanisms

The fourth dimension concerns shifting from FFS, which incents service volume, to alternative payment mechanisms such as capitation or salary which incent service quality and appropriateness. Current evidence supports the thoughtful design of blended payment mechanisms that combine FFS, salary, capitation and pay for performance, to achieve high productivity along with quality and appropriateness [20]. Alternative payment mechanisms can target individual physicians or organizations. At the individual level, physicians can be paid directly through an alternative payment mechanism such as salary. At the organizational level, the organization may be financed based on population needs using per capita payments based on enrolled patients. This mechanism can potentially cover pharmaceuticals, diagnostics, laboratory tests, and care from specialists, public health, rehabilitation centres, long-term facilities and home and community care [42].

#### Dimension 5: patient enrolment

In conventional PC practice, physicians determine which patients they accept, while patients choose which physicians they see. By contrast, patient enrolment (or rostering) requires patients in a defined population or geographical area to register with a specific PC provider. This formalizes a longitudinal relationship between a patient and provider [43] and encourages accountability by defining the population for which the organization or provider is responsible [44]. Patient enrolment can strengthen a population health orientation [43] and assist with determining the appropriate levels of funding for patients. With the support of information technology, enrolment can also assist with management, planning, research and evaluation [43].

#### Dimension 6: patient and community engagement

The sixth dimension focuses on engaging patients and community members in policy, governance and strategy development. Conventional PC practices are almost always physician-led (although corporations operating walk-in clinics with physicians as sub-contractors or tenants are increasingly prevalent in Canada) and involvement of patients or community takes place primarily within the doctor-patient relationship. In PHC, patient and community participation is more broadly defined as community involvement in decisions impacting on care to individuals and communities [34]. Along this dimension, PCR may entail such changes as feedback from patient surveys and advisory committees, development of programs in local communities, and lay governance through community boards [11].

#### Dimension 7: 24/7 Access

A seventh reform dimension looks toward achieving 24/7 access to PC [45]. Lack of after-hours access can increase utilization of walk-in clinics or hospital emergency rooms, thus undermining care continuity, decreasing appropriateness, and, increasing system costs. 24/7 access to care can be enabled through some combination of shift work, after hours clinics, on-call services, telephone advice lines, email communications, patient portals, same-day appointments and telehealth [37].

#### Dimension 8: Information Technology (IT)

The eighth core dimension looks toward the greater use of IT to support clinical practice [46]. Well-designed information management systems such as electronic medical records and electronic health records can support evidence-informed clinical care and decision-making, identification of patients’ care needs, patient engagement and care planning, coordination across the continuum of care, and generating performance measures for healthcare planning, evaluating innovations, and determining resource allocation [17, 20, 47].

#### Dimension 9: system coordination and integration

This dimension looks toward inter-connections between PC and other parts of health and social systems [37, 38]. In Ontario, as in other jurisdictions, the “mainstream” of hospital and doctor care continues to be “siloed” or fragmented with few mechanisms for coordinating care of individuals requiring multiple services from multiple providers [48]; solo PC physicians may have little capacity to follow their patients through the care “pathway.” To enable access to a comprehensive range of services between PC and across sectors, there is a need for interoperable electronic health records, case management and coordination and integration strategies (informal relationships, formal agreements and partnerships, and integrated governance) [49].

#### Dimension 10: Continuous Performance Measurement (PM) and Quality Improvement (QI)

Along with population health, there has been growing recognition of the importance of providing patients with high quality, safe care [50, 51]. Consequently, PM and QI have been recognized as key to transforming PC [46, 52, 53]. This includes tracking progress against population and organizational goals, comparison of performance against both internal and external standards, and identifying opportunities for improvement. Several jurisdictions have supported QI in PC practices by providing educational or practice support [17].

#### Dimension 11: collective governance and leadership

The final dimension involves implementation of effective governance, administration and managerial structures [30] at the local/ regional level with the inclusion of leaders from government, provider associations, front-line practitioners, academics and patients and citizens [17]. Collective governance and leadership can enable population health initiatives and facilitate inter-sectoral collaboration. Australia, New Zealand and the United Kingdom have established governance mechanisms to integrate PC with other services [31, 54]. These mechanisms allow for responding to community needs, negotiating relationships with other health and social services and coordinating or sharing resources, and PM and QI activities [26]. These mechanisms also permit PC organizations and governments to come together and assume collective responsibility and accountability for performance and service delivery [26].

### Part 3: PCR models in Ontario

Since the 1970s, Ontario has implemented an assortment of different PCR models, each bundling different combinations of the 11 core dimensions.

**Fig. 2:**
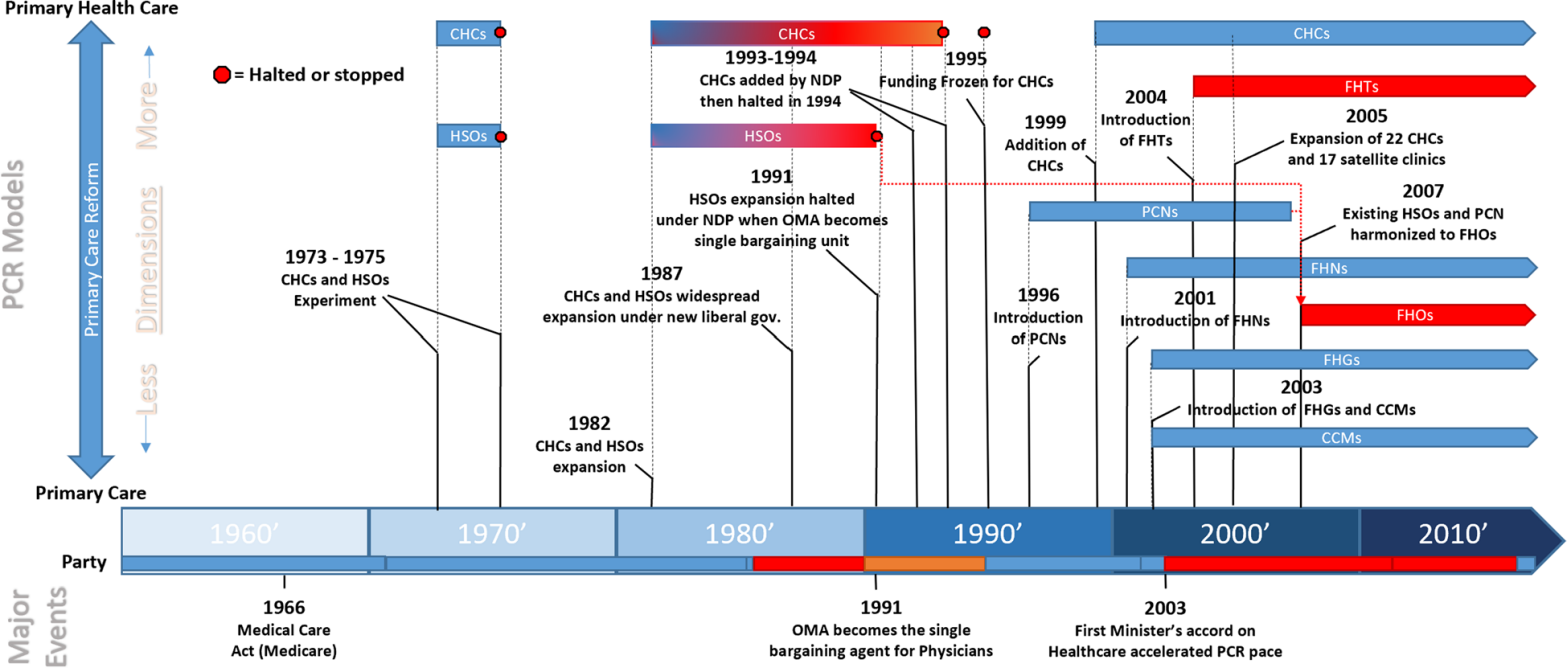
Historical Evolution of PCR in Ontario. *CHC* Community Health Centre, *FHG* Family Health Group, *FHT* Family Health Centre*, FHN* Family Health Network, *CCM* Comprehensive Care Model, *FHO* Family Health Organization

The historical emergence of dominant reform models is shown in Fig. 2; models currently operating in Ontario are subsequently described in the text below. For example, Community Health Centres (CHCs), a model that first appeared in the 1970s, is described in the text since CHCs continue to operate in Ontario. However, Health Service Organizations (HSOs), a group-based model introduced at about the same time, is not described since HSOs have been transitioned to Family Health Organizations (described below).

A first important observation from Fig. 2 is that Ontario has exhibited a considerable penchant for PCR, if judged solely by the proliferation of reform models. Over 4 decades, Ontario has introduced seven different PCR models, with six still operating alongside conventional PC practices. A second observation is that these reform models are highly diverse in terms of their characteristics and change potential: while some, like CHCs (toward the top of the Figure) bundle multiple reform dimensions others, like Comprehensive Care Model (CCM) (seen near the bottom), incorporate few such dimensions.

#### Community Health Centres (CHCs)

As shown in Fig. 2, CHCs were the first PCR model to emerge in the 1970s. From the beginning, CHCs were designed to emphasize illness prevention, health promotion and the social determinants of health for populations or sub-populations in given geographic areas [55, 56]. They also integrated the use of inter-professional teams, 24/7 coverage, community governance and salaried physicians working as employees [34, 56]. However, their impact was limited by an agreement between the government and the medical profession in the 1980s, that allowed the government to establish new CHCs, but only in “underserved” communities [11]. Aboriginal Health Access Centres (AHACs) represent the adaptation of this model to Indigenous (First Nations) communities mostly in Northern Ontario.

#### Family Health Networks (FHNs)

In 2000, the government announced $250 million for expansion of FHNs throughout Ontario. FHNs were based on the pilot Primary Care Network (PCN) model, which have been transitioned to FHOs. The goal of FHNs was to ensure that families had access to convenient, quality health care closer to home. A commitment was made to have 80% of FPs/GPs in FHNs by 2004 [11]. In contrast to CHCs that were *community-led*, FHNs constituted a *physician-governed group* model with a minimum of three physicians. In this model, financing for physicians shifted from FFS to blended capitation/FFS remuneration and physicians were required to enroll patients and provide 24/7 access to care. In January, 2013, the requirement for 24/7 after-hours coverage was removed [57].

#### Family Health Groups (FHGs) and Comprehensive Care Model (CCM)

In April of 2003, FHGs and CCMs were established under a “Re-Opener Agreement” between the provincial government and the medical association [11]. These models followed the limited adoption by physicians of the FHN model. FHGs and CCMs represented a shift back toward more conventional solo, physician-dominated PC practice [11]. A minimum of three physicians would be paid through FFS with additional economic incentives to enroll patients and provide 24/7 care. The CCM represented an even more minimalist reform: it permitted solo FFS physicians access to additional monthly capitation payments, special premiums and incentives for enrolling patients but with no obligation to form a group or provide on-call services [11].

#### Family Health Teams (FHTs) and Family Health Organizations (FHOs)

In 2004, the province announced the creation of 150 FHTs to provide access to PC for 2.5 million Ontarians by 2007–2008 [11]. The goal was to improve access to PHC and change delivery by implementing interdisciplinary teams and promoting the health and wellness of the communities served. The FHO compensation model was also introduced; although similar to the FHN it was financially more lucrative for doctors due to a larger basket of included fee codes and capitation payments [11]. FHOs featured enrolled patients, extended hours, and financial incentives for preventive care and chronic disease management. In order for physicians to participate in the FHT model, which included funding for non-physician providers (e.g., nurse practitioners) as well as capital (e.g., office space), they had to receive compensation under a FHN, a FHO, or the Rural and North Physician Group Agreement (RNPGA).

Table 1 counts the number of core reform dimensions bundled within each of these different models presented in order of their historical emergence. Counts are based on data from the legal contractual agreements between the provincial Ministry of Health and Long-Term Care (which directly funds PC practices with the exception of CHCs which are funded by Ontario’s regional health authorities) and the Ontario Medical Association as of October 2018. In the table, “no” means that a core dimension is not required in the model; “yes,” that it is required; “optional,” that it may be implemented but is not required. Note that even if a particular dimension is not required it may be implemented to some degree within individual organizations, although the extent to which this occurs is not documented. For example, while most FHTs remain physician-governed, some degree of patient and community participation in governance may be achieved through various forms of outreach.

**Table 1:** Core Dimensions by PCR Model

Core Dimensions	CHC	FHN	FHG	CCM	FHT	FHO
1: Population Health Approach	Yes	No	No	No	Yes	No
2: Group Practice Setting	Yes	Yes	Yes	No	Yes	Yes
3: Inter-Professional Teams	Yes	No	No	No	Yes	No
4: Alternative Payment Mechanisms^a^	Yes	Yes	No	No	Yes	Yes
5: Patient Enrolment	No	Yes	Yes	Yes	Yes	Yes
6: Patient and Community Engagement	Yes	No	No	No	Yes	No
7: 24/7 Access to Care	No	No	No	No	No	No
8: Information Technology	Yes	Optional	Optional	Optional	Yes	Optional
9: System Coordination and Integration	Yes	No	No	No	Optional	No
10: Continuous Performance Measurement and Quality Improvement	Yes	No	No	No	Yes	No
11 Collective Governance and Leadership	Yes	No	No	No	No	No
Total Required Core Dimensions	9	3	2	1	8	3

*CHC* Community Health Centre, *FHG* Family Health Group, *FHT* Family Health Centre*, FHN* Family Health Network, *CCM* Comprehensive Care Model, *FHO* Family Health Organization

^a^FHN and FHO (FHT) physicians have the option to receive payment at the individual or group level through capitation payments

The data in Table 1 show that the CHC model incorporates the most core dimensions (nine of 11) including: a population health approach; group practice and inter-disciplinary teams; mechanisms for coordinating with other sectors; IT to maintain individual patient records and assess utilization patterns and outcomes; alternative payment mechanisms (salaried payments for physicians, and population-adjusted global budgets for organizations); patient and community engagement (lay boards and community outreach); PM and QI; and community governance and leadership. However, CHCs are not required to provide 24/7 access or to formally roster patients although individual CHCs may provide after hours care and register patients within their organizations.

FHTs also represent a significant degree of change from conventional PC practice to more expansive PHC. FHTs integrate eight core dimensions (including group practice, inter-professional teams, alternative payment, enrolment, PM and QI, programs based on population health needs, information technology to support email communication with patients, mechanisms for community and patient input). FHTs are also required to participate in health services planning with regional health authorities and to collaborate with other PC practices in their community. However, there are no requirements for system coordination or mechanisms for formal PC governance at the local or regional level.

At the other end of the change spectrum are CCMs, FHGs and FHOs. CCMs integrate only one core dimension (patient enrolment) while FHGs incorporate two dimensions (group practice and patient enrolment). For their part, FHOs integrate three of 10 core dimensions (group practice, alternative payment and enrolment).

**Fig. 3:**
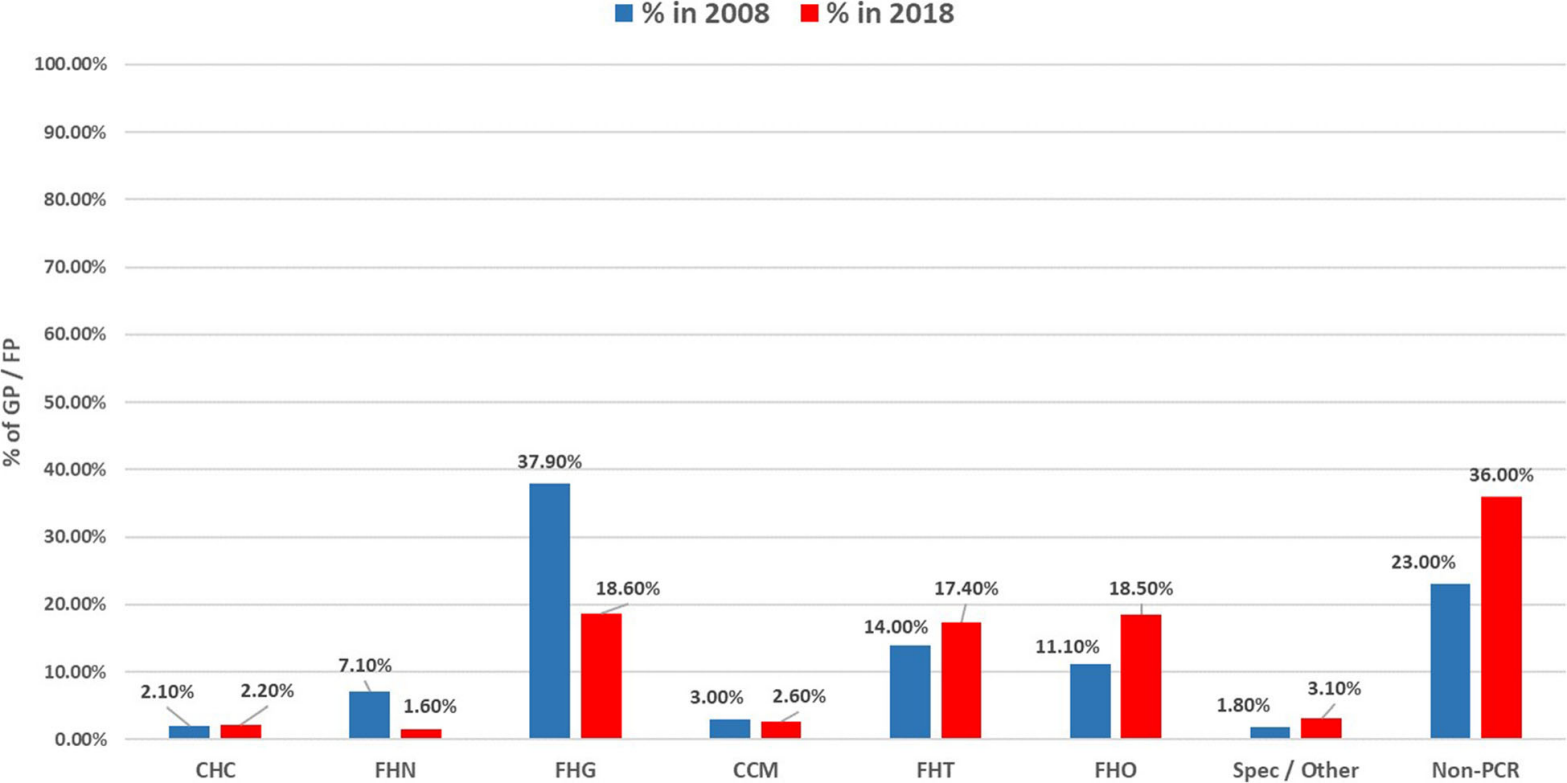
Percentage of FP/GP Participation by PCR Model. *CHC* Community Health Centre, *FHG* Family Health Group, *FHT* Family Health Centre*, FHN* Family Health Network, *CCM* Comprehensive Care Model, *FHO* Family Health Organization

To estimate the impact of these different models, we counted Ontario FPs/GPs participating in each model in 2008 and a decade later in 2018; counts are presented in Table 2 and graphically represented in Fig. 3. This data was obtained from the Ontario Ministry of Health and Long-Term Care. Denominators are numbers of active FPs/GPs in Ontario as of June 2008 (11,042 FPs/GPs) and December 2017 (14,347 FPs/GPs) [58].

**Table 2:** Number of FPs/GPs in PCR Models in June of 2008 and 2018

	Total FPs/GPs	CHC^a^	FHN	FHG	CCM	FHT	FHO	Other Models^b^	Non-PCR Models^c^
# Core Dimensions in 2018		9	3	2	1	8^d^	3	N/A	N/A
FPs/GPs Signed (2008)	11,042	234 2.1%	786 7.1%	4190 37.9%	330 3.0%	1548 14.0%	1221 11.1%	197 1.8%	2536 23.0%
FPs/GPs Signed (2018)	14,347	318 2.2%	232 1.6%	2670 18.6%	361 2.6%	2500 17.4%	2661^e^ 18.5%	519 3.1%	5086 36.0%

*CHC* Community Health Centre, *FHG* Family Health Group, *FHT* Family Health Centre*, FHN* Family Health Network, *CCM* Comprehensive Care Model, *FHO* Family Health Organization

^a^This includes 10 Aboriginal Health Access Centres (AHACs with 20 salaried physicians)

^b^This category includes specialized models that are not mainstream. This includes FPs/GPs in specialized models as of September 2018: 98 Rural and RNPGA; 345 Group Health Centre, Weeneebayko Health Ahtuskaywin, St. Joes, Inner City Health Associates, HIV Groups, Sherbourne Physician Group and Shelter Health Network physicians; 76 in Other APP (Hamilton Maternity and Toronto Palliative Care Associates)

^c^This category includes the number of FPs/GPs in Ontario that are not part of the specified PCR models and Other Models

^d^In FHTs, core dimensions have changed from 2008 to 2018. The 24/7 access requirement has been removed and patient engagement, information technology, QI/PM requirements have been included

^e^There is a total of 5161 FP/GPs in the FHO in which 2500 are in the FHT resulting in 2661 physicians

These data show that in 2008, a majority of Ontario’s FPs/GPs were in practices representing little or no change from conventional PC. FHGs, incorporating just 2 reform dimensions, constituted the most populous reform model accounting for just over a third (37.9%) of FPs/GPs in the province. “Non-PCR models,” effectively conventional PC practices incorporating no reform dimensions, were the second largest category, with just under a quarter (23.0%) of FPs/GPs. CCMs, incorporating a single reform dimension added an additional 3.0%. In total, therefore, these no/low change models accounted for almost two thirds (63.9%) of practicing FPs/GPs. FHNs and FHOs, each bundling 3 reform dimensions, accounted for an additional 18.2% of FPs/GPs. In contrast, CHCs and FHTs, incorporating eight or more reform dimensions, accounted for less than a fifth (16.1%) of FPs/GPs.

The data also show that a decade later, in 2018, the “non-PCR” category had actually expanded by almost half to become the single largest PC practice category, accounting for more than a third (36%) of FPs/GPs. This reflects growth in total GP/FP numbers (from 11,042 to 14,347) and the fact that the provincial government had effectively frozen the expansion of reform models such as FHTs over concerns around rising costs [59]. When combined with no/low change models (CCMs, FHGs) now accounted for 57.2% of GPs/FPs, a slightly lower percentage as compared to 2008. However, this did not translate into significant gains at the other end of the change spectrum: while CHCs and FHTs, both high change models, expanded marginally, they still accounted for just under a fifth (19.6%) of FPs/GPs. In fact, the largest gains are seen in the FHO model incorporating 3 reform dimensions.

**Fig. 4:**
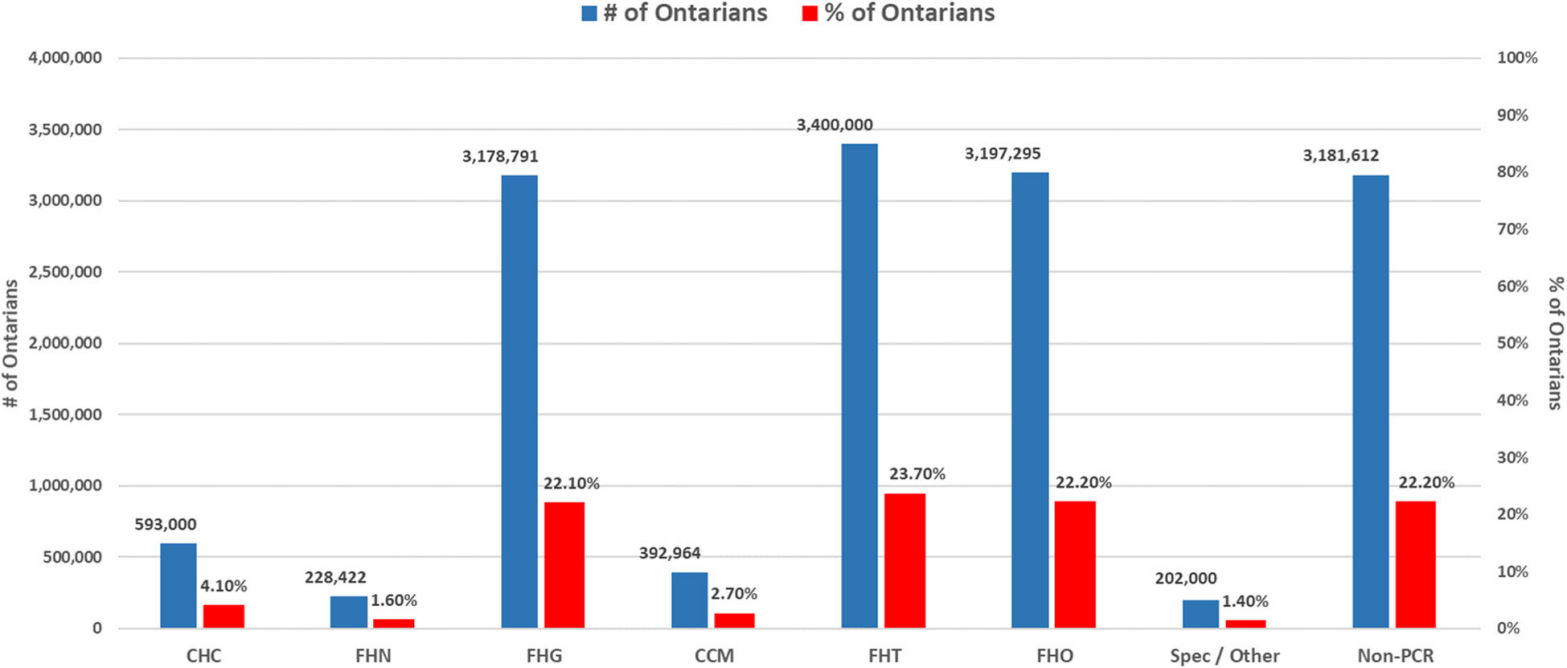
Percentage of Ontarians Participation by PCR Model. *CHC* Community Health Centre, *FHG* Family Health Group, *FHT* Family Health Centre*, FHN* Family Health Network, *CCM* Comprehensive Care Model, *FHO* Family Health Organization

To further assess impact, we counted numbers of patients served by Ontario’s different PC models in 2018. Table 3 and Fig. 4 show numbers of patients enrolled in each PCR model and overall participation rates based on the total population of Ontario as of April 1, 2018 (denominator is 14,374,084 Ontarians) [60].

**Table 3:** Number of Ontarians in PCR Models in June of 2018

	CHC	FHN	FHG	CCM	FHT	FHO	Other Models^a^	Non-PCR Models^b^
# of Core Dimensions	9	3	2	1	8	3	N/A	N/A
# of Ontarians participating	593,000	228,422	3,178,791	392,964	3,400,000	3197295^c^	202,000	3,181,612
% of Ontarians participating	4.1%	1.6%	22.1%	2.7%	23.7%	22.2%	1.4%	22.2%

*CHC* Community Health Centre, *FHG* Family Health Group, *FHT* Family Health Centre*, FHN* Family Health Network, *CCM* Comprehensive Care Model, *FHO* Family Health Organization

^a^Other Models include: RNPGA, GHC, WAHA patients only

^b^This category includes the number of Ontarians that are not rostered or registered in specified PCR models or Other Models

^c^There is a total of 6,597,295 patients in the FHO in which 3,400,000 are in the FHT resulting in 3,197,295 patients

These numbers show that no/low change models (Non-PCR, CCMs and FHGs) accounted for almost half (47%) of Ontario’s total population in 2018. FHGs and FHOs (each incorporating 3 change dimensions) accounted for an additional quarter (23.8%). FHTs and CHCs, models representing a comparatively high change potential, together accounted for another quarter (27.8%) suggesting that these models “hit above their weight” since, as shown earlier, they represented just under a fifth (19.6%) of FPs/GPs in 2018.

## Discussion

As we noted in our introductory remarks, high performing PC is now widely recognized as the foundation of effective and efficient health care systems; even if it does not fully achieve the expansive vision of PHC, PCR is the vehicle for strengthening PC. Many jurisdictions have accordingly embarked on reforms aimed at moving from conventional physician-centred, curative-focused FFS solo practice to more expansive conceptualizations of PHC aimed at achieving broader population health goals through such mechanisms as teams, enrolled populations, alternative payment mechanism, 24/7 access, and collective governance.

Moving in this direction, Ontario, over more than four decades implemented successive waves of PCR and an assortment of different reform models. Judged solely on this basis, one might conclude that it had achieved a considerable degree of change in the organization and delivery of PC. However, as astute observers have cautioned, policy reforms ostensibly aimed at change do not always achieve it, and some “reforms” may reproduce the status quo [61, 62]. Based on our analysis, this seems to be the case in Ontario where in 2018 conventional PC (non-reform) practices accounted for more than a third of all FPs/GPs, and where almost two thirds of FPs/GPs worked in conventional practices or in “no/low reform” models including FHGs and CCMs. Reinforcing this conclusion, such “no/low reform” models served almost half of Ontario’s population during the same year. While clearly some significant change had occurred – higher change models including CHCs and FHTs accounted for about a fifth (18.2%) of all FPs/GPs in the province and about a quarter of all Ontarians (27.8%) in 2018 – overall, movement from PC to PHC was limited. Indeed, the numbers suggest that change was not unidirectional: one of the most marked changes over the decade from 2008 to 2018 was a doubling of the numbers of FPs/GPs working in “non-PCR models” making it the single largest practice category in the latter year.

These findings bring us full circle to our two key points. First, PCR is not a unitary concept or a one-shot deal. Reform is better understood as a multi-dimensional construct with different reform models “bundling” key reform dimensions in different ways. As we have seen in the case of Ontario, while some reform models such as CHCs and FHTs bundle multiple reform dimensions and consequently offer a relatively high potential for change, others like CCMs and FHGs incorporate few dimensions with little movement beyond the status quo.

Second, because PCR aims to change the *delivery* of physician services, it will be inherently tough to accomplish in jurisdictions like Ontario [9, 10]. While highly valued by Canadians because it guarantees universal access to medically necessary care, Canadian Medicare is a *funding* mechanism which, as a result of hard fought historical bargains, leaves control over the organization and conduct of medical practice largely in the hands of physicians themselves. Physician participation in PCR models remains voluntary. Although, as the data confirm, there has been some movement away from FFS toward alternative payment methods in Ontario, governments have often resorted to buying reform by offering additional incentive payments to physicians above and beyond negotiated fee schedules in the hope that they would also buy desired change in PC delivery. However, results have been mixed at best. In addition to driving up costs, there is evidence that capitation/FFS “blends” compared to FFS only have sometimes resulted in less after-hours care, more visits to emergency departments, fewer new patients, and enrolment of low cost patients [63, 64] . Emphasizing again that outcomes may be perverse, a recent study found that financial bonuses designed to incentivize primary care access and minimize primary care visits outside of capitation practices resulted in the unintended consequence of rewarding physicians whose patients made fewer primary care visits, received less after-hours care, made more emergency department visits, and had higher adjusted ambulatory costs [19]. In addition, Ontario’s history of PCR shows that previous attempts to contain lucrative alternative payments (HSO model) resulted in the voluntary migration of physicians back to FFS or other models [11]. This cautions that when change occurs as a response to payment mechanisms, it is not necessarily in desired directions.

With respect to our first point regarding the multi-dimensional nature of PCR, our review of the growing international literature identified 11 commonly identified distinctive reform dimensions; individually and collectively, they push beyond conventional PC practice, toward a more expansive vision of PHC, by emphasizing population health, alternative payment mechanism and rostering, group practice and interdisciplinary teamwork, enhanced access, system coordination, organizational tools such as IT, QI, PM, patient and community engagement, and collective governance and leadership. Here, we do not suggest that these 11 dimensions capture all possibilities; in fact, our own work shows that as the literature around PCR has expanded and matured, additional key dimensions have emerged. In addition, the core dimensions are those that are most frequently identified in the literature.

What our analysis does demonstrate, is the importance of “unpacking” PCR models into their consistuent dimensions to assess how and to what extent they represent movement from conventional PC to more expansive PHC. This analysis allows for an assessment of the degree of progress that has or has not been made with respect to each core dimension, keeping in mind that there may be different degrees of change along different dimensions and that movement may not be unidirectional. While some models such as CCMs and FHGs span only one or two reform dimensions, others, such as FHTs and CHCs span a wider range. In other words, while sharing the title of “reform,” different reform models offer a widely varying potential for change in the organization and delivery of PC.

Our second point, regarding the historical bargains underlying health systems, and the challenges inherent in moving away from these bargains, is equally important since it predicts, in the context of Canadian Medicare, that reform models aimed at substantially changing delivery will be difficult to achieve. Although CHCs and FHTs represent exceptions, much reform effort in Ontario has come down to tinkering with payment methods (including alternatives to FFS and financial incentives) in the hope that this will lead to desired change in organization and delivery. However, as the data show, high change models like CHCs and FHTs remain outliers, while no/low change models, including conventional PC practice itself, represent the mainstream. Indeed, over the decade between 2008 and 2018, it may be concluded that PCR efforts suffered a setback as the number of FPs/GPs practicing in “non-PCR models” doubled.

In addition to establishing a solid foundation for analyzing what has transpired in Ontario, we suggest these two points – “unpacking reform” and assessing them in relation to historical “bargains” underlying health care systems – are also key to conducting meaningful comparative analysis of reforms across jurisdictions, whether within Canada or internationally. Not only do they point to the importance of assessing the extent to which different reforms offer movement toward the goal of PHC, they highlight the need to see reform not just as a technical issue, but as a political project likely to be contested since it can disrupt historical patterns and relationships, particularly, physician control over the organization and delivery of PC. For example, Canada has witnessed many recommendations from expert commissions over 30 years to move PC under regional authority as a way of aligning it more closely with other health services and enhancing accountability for performance and service delivery [8, 65, 66]. In 2016, the Ontario government planned to have FHTs fall under its the regional health authorities [67]. However, few such plans have ever moved beyond the drawing board, largely because medical associations continue to insist that any such changes must be purely voluntary.

The limitations of this study are that the analysis is based on a literature review and analysis of quantitative data on participation on FPs/GPs and Ontarians. The inclusion of key informant interviews involved in the development and implementation of PCR models could have further validated the research findings. In addition, data was not available on the participation of Ontarians in 2008, which prevented the analysis of 10-year trends. Nevertheless, this study is the first to evaluate the pace of change by conducting a detailed analysis of the key elements that are part of each PCR model in Ontario.

## Conclusions

The framework developed in this paper is meant to demonstrate that the pace and direction of reform cannot be evaluated without “unpacking” reforms along multiple dimensions. Assessment of reform models against these core dimensions demonstrate that there has been little substantive change in the organization and delivery of primary care in Ontario over 10 years. This paper provides a conceptual framework that can be used to assist decision-makers, academics and health care providers in all jurisdictions in assessing the pace of change in the PC sector. It also serves to provide perspective on the dimensions that are more or less likely to be implemented based on the institutional structures and relationships embedded in a jurisdiction’s health care system.

## Data Availability

The data analyzed during the current study was obtained from the Ontario Ministry of Health and Long-Term Care in Canada. All the data is presented in the published article. The data is not published on a regular basis but is available by government by request. This data is available from the corresponding author on reasonable request.

## Abbreviations

AHACs: Aboriginal Health Access Centres
CCM: Comprehensive Care Model
CHC: Community Health Centre
EMR: Electronic Medical Record
FFS: Fee-for-Service
FHG: Family Health Group
FHO: Family Health Organization
FHT: Family Health Team
FPs/GPs: Family Physicians/General Practitioners
HSO: Health Service Organization
IT: Information Technology
P/T: Provincial/Territorial
PC: Primary Care
PCR: Primary Care Reform
PM: Performance Measurement
QI: Quality Improvement
RNPGA: Rural and North Physician Group Agreement
